# Ramadan Nutritional Strategy: Professional Soccer Player Case Study

**DOI:** 10.3390/nu14030465

**Published:** 2022-01-21

**Authors:** Alejandro Martínez-Rodríguez, Laura Miralles-Amorós, Manuel Vicente-Martínez, Nuria Asencio-Mas, Rodrigo Yáñez-Sepúlveda, María Martínez-Olcina

**Affiliations:** 1 Department of Analytical Chemistry, Nutrition and Food Science, Faculty of Sciences, University of Alicante, 03690 Alicante, Spain; laura.miralles@ua.es (L.M.-A.); niam1@gcloud.ua.es (N.A.-M.); maria.martinezolcina@ua.es (M.M.-O.); 2 Alicante Institute for Health and Biomedical Research (ISABIAL Foundation), 03010 Alicante, Spain; 3 Faculty of Health Science, Miguel de Cervantes European University, 47012 Valladolid, Spain; mvmartinez11006@alumnos.uemc.es; 4 Escuela de Educación, Pedagogía en Educación Física, Universidad Viña del Mar, Viña del Mar 7055, Chile; rodrigo.yanez@uvm.cl

**Keywords:** nutrition, sport, athletic performance, sports supplements, body composition, dietary tools

## Abstract

The period of Ramadan induces changes in the usual eating patterns of individuals. During this period, Muslims must abstain from drinking and eating from dawn to dusk. Therefore, some research conducted on professional soccer players has observed that during and/or after Ramadan, performance, running speed, agility, dribbling speed, and endurance and/or skill performance in athletic events may be negatively affected by Ramadan intermittent fasting (RIF). The objective of this study was to analyze the influence of a dietary plan during RIF on performance and body composition in a professional soccer player. A 20-year-old elite player (86.0 kg, 188.5 cm) followed a dietary-nutritional plan with an isocaloric diet and was supplemented with glycerol. The athlete’s strength and power in the lower limbs was assessed by performing a countermovement jump (CMJ) and Abalakov vertical jump (ABK) before and after Ramadan. After nutritional planning, the patient’s body composition improved in terms of fat loss (6.61 to 5.70%) and muscle mass gain (50.26 to 51.50%). In addition, this translated into improvements in performance tests, both in the CMJ (36.72 to 40.00 cm) and ABK (39.16 to 49.34 cm). In conclusion, during a period of fasting, personalised nutritional planning and an appropriate supplementation and rest protocol can improve the body composition and performance of soccer players.

## 1. Introduction

The period of Ramadan induces changes in the usual dietary patterns of individuals; during this period, Muslins must abstain from drinking and eating from sunrise to sunset [1]. According to the Holy Quran, it takes place in the ninth month of the Islamic calendar, and, as a lunar month, it lasts between 29 and 30 days [2].

According to the Federation International Football Association (FIFA), soccer has promoted fair play, heterogeneity and fairness and is an internationally recognized sport [1]. The month of Ramadan often coincides with the calendars of many soccer leagues [1]. Researchers have proceeded to conduct studies on adult and youth Muslim soccer players because of their commitment to their faith and the game. It has been shown that, during and/or after Ramadan, performance, running speed, agility, dribbling speed, and endurance and/or skill performance in athletic tests can be negatively affected by Ramadan intermittent fasting (RIF) [3,4].

In the same way, sleep duration is compromised, as dinners and breakfasts are usually early during Ramadan [5,6]. Indeed, by altering the sleep-wake cycle of circadian rhythms, a modification in anticipation and adaptation to environmental changes occurring during the day may occur in the metabolism and cardiovascular system [2]. Therefore, biological rhythms and how they affect human performance must be taken into account, as some factors, such as hepatic and muscular glucogen stores, fluid stores, decreased blood glucose levels, increased uric acid and the risk of dehydration during prolonged physical activity, could negatively impair athletic performance [7].

Currently, there are no studies evaluating the effects of Ramadan that intervene at the dietary-nutritional level, so there are still no adapted plans for practitioners practicing RIF [1,8]. However, the variation in total energy intake before and after Ramadan has been evaluated [6,9,10]. In these investigations, it has been observed that the intake was insufficient to meet the needs of the athletes and, in addition, the estimated total daily energy intake and the relative proportions of carbohydrate, fat and protein in the diet did not change during Ramadan in the participants [6,9,10].

Physical performance tests such as counter movement jump (CMJ) and Abalakov jump are often used in these investigations to assess recovery from baseline levels and serve to measure power and fatigue [11,12]. Results for CMJ have been inconclusive in the literature [8]. Rebaï et al. [13] observed significant increases in CMJ during Ramadan; the increases were significant during versus before Ramadan for the group that reduced their training load. However, some of the studies found do not evaluate the effect of RIF on Abalakov’s jumping tests, with free arm jumps being predominant in this sport [9,10,13].

Another aspect that influences sports performance and can be affected by RIF is body composition. In soccer, it is important to control body fat, as optimal fat levels allow players to move more efficiently during training and matches [11,14]. Further relevant is the lean mass compartment, specifically muscle mass, because insufficient or excessive training loads can impact the physique, affecting performance, as well as speed, strength, power and injury risk [15].

Although inconsistent findings on body composition for RIF have been observed in the scientific literature [1,8], some studies use data obtained from bioimpedance or the summation of 4 folds [6,9]. In addition, other research proposes, but does not carry out, the complete profile of 5 components: skin, body fat, muscle mass, bone tissue and residual mass. This model allows to get a photographic shape of the athlete’s profile [16].

Another aspect that lacks research so far is the medio somatotype and the Heath and Carter anthropometric method classification [17] for Muslim RIF performers.

In order to respond to all these gaps in the scientific literature, the main objective of this research was to describe the effects of RIF with personalized dietary-nutritional planning on the athletic performance and body composition of an elite soccer player. The initial hypothesis was that performance would be negatively affected by the month of Ramadan.

## 2. Materials and Methods

### 2.1. Study Design

A case report was used to analyse the influence of RIF on performance and body composition in a professional soccer player. In this design, an initial measurement prior to the intervention (start) and a measurement after the intervention (final) was conducted.

The participant was informed about the possible risks and discomforts that could arise and was asked to complete a health history questionnaire and sign a consent form. The research strictly followed the Declaration of Helsinki (Edinburgh review in 2000), and the procedures were also in accordance with the recommendations of the EEC Good Clinical Practice (document 111/3976/88 of July 1990). The subject signed an informed consent to participate in the study after receiving all the information. All procedures were previously approved by the Ethics Committee of the University of Alicante (UA-2021-03-11).

### 2.2. Participant

The participant was a 20-year old African soccer player since he was 5 years old, having now 15 years of experience. He has been playing soccer at a professional level, for the team in La Liga for the last two seasons. He usually trains 12 h per week and his only dedication is soccer. He is healthy, does not take any medication, has never undergone surgery and has an optimal blood biochemistry.

### 2.3. Data Collection

#### 2.3.1. Body Composition

Body weight was measured early in the morning, fasting and with minimal clothing, using a Tanita BC-545n, (Tanita Corporation, Arlington Heights, IL, USA) to the nearest 0.1 kg. Standing height without shoes was measured using a Seca 213 stadiometer (Seca, Hamburg, Germany) to the nearest 0.1 cm. To minimize the potential source of Bioimpedance systems (BIA) related to total body weight and height, body composition assessment was performed by a level three anthropometrist following the International Society for the Advancement of Kinanthropometry (ISAK) recommendations [18]. All measurements were performed at the same location, at room temperature and under baseline conditions. The technical error of measurement for perimeters, circumferences, lengths and heights was less than 1% and less than 5% for skinfolds.

Anthropometric measurements were performed following the complete profile of ISAK II methodology [18]. Skinfolds, girths, lengths and breadths were measured with a caliper, flexible metallic tape, segmometer and pachymeter, respectively (Holtain, Crymych, UK).

Bone and muscle mass were obtained through Rocha’s equation [19] and Lee’s formula [20], respectively. Fat mass was estimated using Carter’s formula [21], Faulkner formula [22] and Withers’ equation [23]. Residual mass was calculated from the difference between the total body weight minus the sum of the bone, muscle and fat masses. According to the Spanish Committee of Kinanthropometry, these methods are the most suitable for high performance players [24].

Somatotype components were calculated from the assessment of different body compartments, including fat mass for endomorphy, muscle mass for mesomorphy, and leanness and relative bone linearity for ectomorphy. The differences between each individual somatopoint with respect to the mean value was calculated using the somatotype attitudinal mean (SAM) [25,26].

Proportionality analyses were performed using the Phantom stratum [19]. Each variable was fitted to the Phantom size using the z-score. The z-values have a mean of 0, so a z-value of 0.0 indicates that the given variable is proportionally equal to the Phantom; a z-value greater than 0.0 means that the subject is proportionally greater than the Phantom for that variable; and, conversely, a z-value less than 0.0 shows that the subject is proportionally less than the Phantom for the variable [19].

#### 2.3.2. Performance Measures

As indicators of global strength, the countermarch jump (CMJ) and the Abalakov jump test [27] were performed. In the CMJ test, the participant performed a maximal vertical jump starting from a standing position, without allowing the arms to swing, and bending the knees 90°. The participant performed several familiarisation trials prior to the test [28]. A contact platform (Optojump Next Microgate, Bolzano, Italy) was used to measure the CMJ. Three measurements were performed with 30 s recovery in between. The flight time was used to calculate the jump height. The best jump was used for subsequent analysis [29,30].

The Abalakov jump test followed the same protocol. The participant also performed 3 countermovement jumps with 30 s rest between jumps, on a platform with an optical (infrared) data collection system (Optojump Next Microgate, Bolzano, Italy) to calculate the Abalakov jump height [31]. The player had to stand up and perform a 90° knee flexion followed by the fastest extension to reach the highest possible jump height. Of the three results, the best one was used for statistical analysis.

### 2.4. Nutritional Intervention Protocol

#### 2.4.1. Nutrient Intake

For the dietary-nutritional intervention, quantitative estimates were made of total energy expenditure based on basal metabolism, using the Harris–Benedict formula [32] and corrected body weight. Physical activity expenditure was estimated from standardized factors [33]. The proposed diet was based on an isocaloric intake following the recommendations for elite soccer players [34].

Table 1 shows the values of macronutrients and vitamin B12 provided by the dietary-nutritional guideline established according to the recommendations for elite soccer players. Fiber intake was around 25 g per day and total cholesterol was less than 200 mg per day. The software used in the elaboration of the diet was Dietopro (Dietopro, Valencia, Spain) [35].

Because the time of ingestion is different than usual during the RIF, a schedule was established with SK for each of the different meals (Table 2).

#### 2.4.2. Supplementation

Glycerol (1,2,3-propanetriol) is produced from glucose, protein, pyruvate, triacylglycerols and other glycerolipid metabolic pathways and is a binding metabolite in numerous pathways [27].

During RIF, glycerol is the only source of gluconeogenesis, as glycogen stores are depleted within two days of fasting [28]. Considering its role as an energy substrate, glycerol could effectively improve sports performance [28]. The combined ingestion of glycerol and fluid has been used to increase body water volume, thus maintaining hydration by reducing the rate of water elimination from the kidneys [27]. The intake protocol is shown in Table 3.

## 3. Results

### 3.1. Body Composition

The soccer player remained weight-stable throughout the study period (the initial weight was 86.1 kg, while at the end of the intervention the weight reached 85.9 kg). The athlete claimed to have followed the entire nutritional plan, based on 3400 kcal, 4.6 g/kg/day of carbohydrates and 2.5 g/kg/day of protein.

Table 4 shows the results of the body composition assessment between the beginning and the end of the RIF. It can be observed that both weight and fat mass percentage decreased after the intervention. However, muscle mass increased (from 50.26 to 51.50%). The somatotype presented is ecto-mesomorphic, where mesomorphy predominates and ectomorphy is higher than endomorphy.

With regard to skinfolds, the triceps (4.5 to 3.5 mm), subscapular (8.2 to 7.6 mm), biceps (3.4 to 2.5 mm), supraspinal (4.5 to 4.2 mm), abdominal (5.8 to 4.8 mm), thigh (6.3 to 6.1 mm) and leg (4.3 to 2.8 mm) skinfolds decreased, while the triceps (4.3 to 2.8 mm) skinfold decreased, (5 to 4.2 mm), abdominal (5.8 to 4.8 mm), thigh (6.3 to 6.1 mm) and leg (4.3 to 2.8 mm) decreased, while the iliac crest crease increased (6.1 to 6.4 mm).

Differences were observed in the different variables for anthropometric dimensions and proportionality profile. The results for Z-perimeter calf (0.06 to 0.20), Z-perimeter thigh (−0.08 to −0.25), Z-perimeter hip (−0.95 to −1.72), Z-perimeter waist (−1.17 to −1.20), Z-perimeter forearm (1.96 to 2.28), Z-perimeter flexed arm (2.49 to 3.15) and Z-perimeter relaxed arm (1.98 to 2.06) were higher after the intervention. However, the Z-perimeter mesosternale decreased from −0.28 to −0.20.

### 3.2. Sport Performance

Regarding sport performance results, lower limb power measured with the CMJ and Abalakov jumps tests shows a considerable improvement in performance after the RIF and the dietary-nutritional intervention performed. The variation of the Abalakov jump was from 39.16 to 49.34 cm, while the countermovement jump improved from 36.72 to 40 cm.

## 4. Discussion

The main objective of this research was to evaluate body composition, physical condition (through different physical tests) and a personalized dietary-nutritional plan in a professional soccer player at the beginning and at the end of the Ramadan period.

Differences in daily energy intake among different studies could be due to dietary habits, cultural customs, biopsychosocial environment, and geographical differences of the study participants. The present findings demonstrate that the estimated total daily energy intake was not affected by Ramadan fasting, since the player followed the dietary pattern, where the nutritional plan was adapted to the fasting schedule [29,30,31].

In terms of body composition, the player presented lower initial characteristics of muscle mass and higher fat mass compared to the end of the study. This can also be seen in the measurement of skinfolds, where all decreased, except for the iliac crest. So far, no study has analyzed these parameters in such depth. Only one study by Durnin and Womersley et al. [36] analyzed the sum of 4 skinfolds (triceps, biceps, subscapular and suprailiac); it was observed that there was no significant difference after the RIF period [37]. Another investigation by Meckel et al. [9] found a slight increase in skinfolds. These studies did not include the implementation of a personalized dietary plan, so the results may be due to an inadequate diet.

It should be noted that this is the first study to investigate the effect of a dietary-nutritional planning adapted to Ramadan on lower limb physical performance variables and 5-component body composition. This study consists of the CMJ and Abalakov jump tests, being the first study to evaluate Abalakov in a Muslim professional athlete. In reference to the scores, all were higher after the intervention. Therefore, the player had better lower body power at the end of the study than at the beginning. These results at first indicate an increase in physical performance tests, however, they are different from those obtained in previous studies, as some studies showed that Ramadan fasting decreases physical performance [9,37], while others revealed no effect [3,4,6].

Furthermore, since Abalakov tests can be used as a performance indicator and as part of the selection process of youth soccer players for national teams [38], future research should include this test in its variables.

On the other hand, some researchers [27] underline the importance of the qualities of glycerol for success in aerobic and anaerobic activities, emphasizing the effect on performance, since it can be used as an energy substrate. The findings of such supplementation evidence its relevance and usefulness in the athlete population, since it has been observed that hypohydration decreases maximal aerobic power [27]. Therefore, the effects of such supplementation could have had a positive impact on the performance tests of the subject included in the present study, following the same line as other investigations [39].

Existing scientific evidence shows that adequate hydration and nutrition are essential to improve performance in soccer during Ramadan periods [1,40]. It should be borne in mind that soccer is a high-intensity intermittent sport played in hot environments, so there is an increased risk of dehydration, with sweating rates in elite players of 1–2.5 L/h [39]. Therefore, both physiological function and cognitive and athletic performance are compromised. To maximise the effects of training during Ramadan and better adapt to the conditions presented, it is essential to make appropriate dietary choices, ensuring optimal energy intake during periods of high-intensity, long-duration training [1].

The results of this case study should be interpreted with caution, as they are based on the performance of a single athlete using an experimental protocol (*n* = 1) in which neither the participant nor the researchers were unaware of the project during the intervention phase. In fact, only one set of performance tests was conducted at the beginning and end of the intervention. Future research will consider the previously mentioned limitation. It is recommended that researchers in the field provide more detailed information on fitness assessment, body composition and dietary habits in future studies on professional soccer players and the RIF, as research is still scarce.

## 5. Conclusions

During a fasting period such as Ramadan, an optimal and personalized nutritional planning according to the demands and needs of each athlete, accompanied by a supplementation of glycerol and rest protocol, allows for improvements on body composition and lower limb performance indicators in soccer players.

## Figures and Tables

**Table 1:** Dietary nutrient intake.

Calories (Kcal)	3432.1
Carbohydrates (g)	398.1
Proteins (g)	219.4
Fat (g)	112.7
Vitamin B12 (mg)	17.3
Carbohydrates (g/kg/day)	4.6
Protein (g/kg/day)	2.5
PFA/MFA	1.2

Kcal = kilocalories; g = grams; kg = kilograms; PFA = polyunsaturated fatty acids; MFA = monounsaturated fatty acids.

**Table 2:** Meal times.

Intake	Time of Day
Breakfast	5:30–6:00
Snack	20:00
Dinner	22:30
Snack	0:30

**Table 3:** Glycerol intake protocol.

Time	Glycerol (g)	Liquid (L)
1–3 days	25	0.5
4–6 days	50	1
7–10 days	75	1.5
11–final	100	2

g = grams; L = liters.

**Table 4:** Body composition results.

Body Composition	Start	Final
Weight (kg)	86.1	85.9
Height (cm)	188.5	188.5
BFM Carter (%)	3.80	3.30
BFM Faulkner (%)	9.30	8.90
BFM Withers + Siri (%)	6.61	5.70
Muscle mass (kg) Lee 2000	43.27	44.20
Muscle mass (%) Lee 2000	50.26	51.50
Bone mass (kg) Rocha	13.96	14.01
Bone mass (%) Rocha	16.21	16.31
Residual mass (kg)	23.18	22.81
Residual mass (%)	26.92	26.55
Endomorphy	1.38	1.16
Mesomorphy	5.46	5.98
Ectomorphy	2.67	2.69

BFM: Body Fat Mass; kg = kilograms; cm = centimeters; % = percentage.

## Data Availability

The data presented in this study is available on request from the corresponding author. The data are not publicly available due to is personal health information.
